# Utilizing Multifaceted Approaches to Target Drug Delivery in the Brain: From Nanoparticles to Biological Therapies

**DOI:** 10.7759/cureus.68419

**Published:** 2024-09-01

**Authors:** Sameenkhan Anwarkhan, Jebastin Koilpillai, Damodharan Narayanasamy

**Affiliations:** 1 Department of Pharmacy, Sri Ramaswamy Memorial (SRM) Institute of Science and Technology, Kattankulathur, IND; 2 Department of Pharmaceutics, Sri Ramaswamy Memorial (SRM) Institute of Science and Technology, Kattankulathur, IND

**Keywords:** viral vectors, biological therapies, nanotechnology, neurological disorders, blood-brain barrier (bbb)

## Abstract

The blood-brain barrier (BBB) poses an important obstacle to treating neurological disorders because it limits the entry of therapeutic agents into the central nervous system (CNS). Surmounting this barrier is crucial for delivering drugs effectively and targeting precise areas of the brain affected by conditions like Parkinson's disease, Alzheimer's disease, and brain tumors. This review examines the diverse strategies employed to enhance brain targeting, including nanotechnology, viral vectors, and biological therapies. Nanoparticles, liposomes, and dendrimers offer promising approaches for encapsulating drugs and facilitating their transport across the BBB. Viral vectors, such as adeno-associated viruses, demonstrate high transfection efficiency for gene therapy applications in CNS diseases. Biological therapies, including stem cell transplantation and neuromodulation techniques, can potentially restore normal cellular function and treat genetic disorders. Challenges such as BBB permeability, safety concerns, and regulatory considerations are discussed, along with future perspectives on precision medicine, noninvasive delivery methods, and biomarker discovery. By addressing these challenges and embracing innovative approaches, the field of brain drug targeting aims to transfer the way that neurological illness is treated and improve patient outcomes.

## Introduction and background

The blood-brain barrier (BBB) poses a formidable opposition to effectively treating many brain diseases because it limits the passage of cells and molecules between the bloodstream and the brain. Successfully overcoming this barrier is essential for delivering pharmacologically active molecules to targeted sites within the central nervous system (CNS). Multiple strategies have been suggested to enhance drug delivery through BBB. These include locally bypassing the barrier with direct injections or nasal applications of drugs and enhancing overall delivery through the bloodstream [1].

An emerging frontier in pharmaceutical research entails the development of brain-targeted drug delivery systems, which hold particular significance in addressing CNS disorders while surmounting the formidable BBB. This barrier, primarily constituted of brain microvascular endothelial cells endowed with tight junctions (TJs), poses formidable difficulties to the passage of therapeutic compounds from the systemic circulation into the brain parenchyma [1]. To confront this challenge, innovative strategies such as nanocarrier-based drug delivery systems have been devised, offering the capability to encapsulate drugs and augment their transport across the BBB. Moreover, molecular targeting methodologies employing ligands specifically tailored to receptors expressed on brain endothelial cells present a promising avenue for facilitating enhanced drug penetration into the CNS [1]. These advancements are significant for elevating the therapeutic efficacy and mitigating off-target effects in treating neurological conditions, encompassing neurodegenerative diseases and brain malignancies. Nevertheless, continued research and development endeavors are imperative to refine the design and performance of brain-targeted drug delivery systems, thereby ensuring their safety, efficacy, and translational viability.

A tightly regulated microenvironment is vital for the normal operations of the CNS. A biological membrane at the blood-brain interface effectively separates the brain and the rest of the body. Paul Ehrlich initially discovered this barrier when he observed that a dye injected peripherally failed to stain brain tissue [2]. Subsequent experiments by Ehrlich's colleague, Goldmann, using the same dye in cerebrospinal fluid (CSF), further validated this finding by demonstrating selective staining of brain tissue without leakage into the surrounding areas [2].

These biological membranes are formed by distinct cells at three critical interfaces: the BBB, arachnoid barrier, and blood-cerebrospinal fluid barrier (BCSFB). In most mammals and other creatures with a fully developed CNS, the cerebral capillaries that pierce the brain and spinal cord form the BBB. These capillaries are lined by microvascular endothelial cells. It is recognized as the primary interface for blood-brain swap and is estimated to have a combined surface area per average adult ranging from 12 to 18 m^2^. The BBB is crucial for guarding the brain parenchyma from blood-borne substances and is a major barrier that restricts the passage of drugs and other external compounds into the CNS.

The second barrier, known as the BCSFB, is established by the epithelial cells of the choroid plexus. These cells regulate the secretion of CSF into the brain's ventricular system. Conversely, the interstitial fluid (ISF), which makes up most of the brain's extracellular fluid, is partially formed through secretion across the capillary endothelium of the BBB. The ISF interacts freely with CSF at multiple sites, contributing an estimated 10%-60% of CSF volume. Beneath the dura mater lies the avascular arachnoid epithelium, recognized as the third barrier. The dura mater envelops the CNS, sealing off the extracellular fluids of the CNS from those in the rest of the body. Compared to other barriers, its contribution to the interchange of blood and cerebral matter is negligible due to its avascular nature and very limited surface area [3].

The formulation of drug delivery systems targeting the brain necessitates meticulous consideration of various factors, including the physicochemical properties of the therapeutic agents, carrier materials, and strategies for overcoming the BBB. Nanocarrier-based formulations, such as liposomes and polymeric nanoparticles, offer promising platforms for encapsulating drugs and enhancing their transport across the BBB [1]. However, challenges persist in achieving optimal drug loading capacity, stability, and controlled release kinetics within the complex CNS microenvironment. Furthermore, the design of targeting ligands capable of selectively binding to receptors expressed on brain endothelial cells presents a formidable obstacle, necessitating precise molecular engineering and validation.

Addressing these challenges requires interdisciplinary collaboration among pharmaceutical scientists, chemists, biologists, and clinicians to elucidate fundamental mechanisms governing BBB permeability and optimize formulation strategies accordingly. Moreover, translating preclinical formulations to clinical applications mandates rigorous assessment of safety profiles, pharmacokinetics, and biodistribution patterns to ensure efficacy and minimize potential adverse effects [3]. Despite these obstacles, advances in nanotechnology, biomaterials, and targeted drug delivery show great promise in transferring the treatment of neurological disorders. They aim to enable precise, efficient, and safe delivery of therapeutic compounds to the brain parenchyma.

## Review

The BBB challenges and strategies to overcome the challenges

The BBB serves as a robust protector of the CNS, carefully controlling the movement of molecules into and out of the brain to preserve its intricate environment. Made up of specialized brain microvascular endothelial cells, the BBB exhibits unique structural and functional characteristics distinct from those found in peripheral blood vessels. Among its notable characteristics are TJs that tightly seal the intercellular spaces, effectively preventing the uncontrolled movement of polar molecules between the bloodstream and the brain parenchyma.

TJs are just one component of the BBB; other components include efflux transporters like breast cancer resistance protein (BCRP) and P-glycoprotein (Pgp), which actively transfer potentially hazardous substances out of endothelial cells to reduce their exposure to the CNS. Moreover, the presence of drug-metabolizing enzymes, such as cytochrome P450, within endothelial cells adds to the metabolic barrier by aiding in the biotransformation and clearance of drugs. While the BBB plays a crucial role in protecting the CNS, it also presents a significant challenge for drug delivery in treating neurological disorders [3]. Current invasive methods, such as intrathecal drug administration, are associated with risks and limitations, necessitating the exploration of noninvasive strategies to breach the BBB.

Passive transcytosis emerges as a promising approach, leveraging both paracellular and transcellular pathways to facilitate the transport of molecules across the BBB. By modulating TJ proteins and employing nanoparticles engineered with brain tumor-targeting ligands, researchers aim to enhance BBB penetration and achieve targeted drug delivery.

Intranasal administration offers another noninvasive avenue for brain targeting, capitalizing on neuronal pathways for drug transport. Nanoparticle formulations optimized for intranasal delivery exhibit improved brain distribution and therapeutic efficacy in various neurological disorders, promising rapid and effective drug delivery to the CNS.

Active targeting strategies, such as ligand conjugation, involve the attachment of ligands with high specificity for receptors expressed on brain endothelial cells. Nanoparticles decorated with ligands such as antibodies and peptides demonstrate enhanced brain accumulation and therapeutic outcomes in preclinical studies, offering a targeted approach to drug delivery.

**Figure 1:**
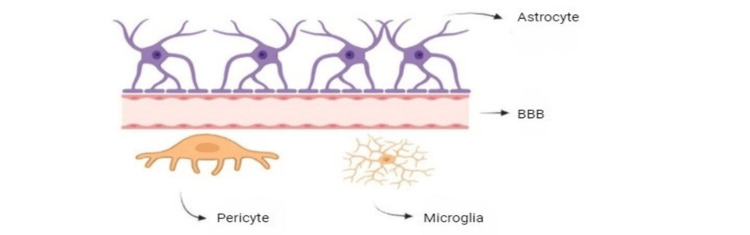
Blood-brain barrier BBB: blood-brain barrier Image credit: This image is the original work of the author Sameenkhan Anwarkhan

Moreover, the utilization of cell membrane coating strategies represents a novel and innovative approach to overcome the BBB. By incorporating membranes derived from various cell types, including red blood cells, brain tumor cells, and immune cells, researchers aim to capitalize on the unique functionalities and molecular interactions of these membranes to facilitate targeted drug distribution to the brain. Overall, the development of noninvasive strategies to overcome the BBB presents a promising frontier in drug delivery for neurological disorders, offering new hope for effective treatments and improved patient outcomes. The detailed internal structure of the BBB is presented, highlighting its various components and intricate design in Figure 1.

Advancements in vitro models for testing BBB functionality

Testing the BBB using in vitro models typically involves a simplified setup consisting of cerebral endothelial cells cultured on a semipermeable contribute under static conditions. Primary brain endothelial cells, isolated from various animal species including bovine, mouse, porcine, rat, and human, or immortalized cell lines, are commonly used for these models. While primary cells offer physiological relevance, their use can be limited by availability, cost, and susceptibility to contamination. On the other hand, immortalized cell lines provide greater reproducibility and accessibility, making them a more convenient option for many studies. In addition to endothelial cells, coculture models incorporating astrocytes and pericytes are widely utilized due to their roles in BBB development and modulation of endothelial cell function. Astrocytes, despite not being in direct contact with the endothelial cells in the neurovascular system, significantly influence interendothelial junctions and transporter expression, particularly during adulthood. Although less well-characterized, pericytes contribute to BBB integrity through various functions such as contractility, immune response, and angiogenesis. Because stem cells may develop into cerebral endothelial cells, they are a good starting point for in vitro models of the human BBB. Induced pluripotent stem cells and embryonic stem cells offer morally acceptable substitutes with the capacity for endless self-renewal and differentiation into distinct kinds of brain cells. Neural stem cells in adult brains offer multipotency and the potential for more complex neurovascular unit models. Mesenchymal stem cells are easily isolated and expanded, exhibiting similar properties to pericytes and offering the potential for brain disease treatment [4].

To facilitate drug transportation studies, culture setups have evolved, with the transwell system being a common choice. This system utilizes a microporous semipermeable membrane to separate vascular and parenchymal compartments, allowing for linear kinetic studies of transport. However, limitations such as lack of physiological three-dimensional structure and shear stress exposure affect endothelial differentiation and BBB phenotype maintenance. Overall, in vitro models of the BBB give expensive tools for drug testing and disease modeling, with ongoing efforts to enhance their physiological relevance and predictive capabilities.

Advancements in in vivo models for testing BBB functionality

Understanding the BBB and its dynamic nature presents challenges in developing effective in silico or in vitro models for predicting drug transport to the CNS. Consequently, in vivo testing remains the gold standard for evaluating CNS drug delivery. These tests measure two important parameters: the extent of distribution inside the CNS (brain distribution volume or partition coefficient, Kp, and brain) and the rate of chemical crossing into the brain (BBB permeability-surface area product). Since free drug concentration is highly correlated with pharmacodynamics models, it is imperative to distinguish between free or unbound drug concentration and total drug concentration in the brain and serum. Methods such as equilibrium dialysis or ultrafiltration can determine the unbound fraction of drugs, enabling the calculation of free concentration (Cu). As an alternative, Cu in the brain may be measured directly by in vivo cerebral microdialysis. The area under the curve (AUC)blood/AUCbrain ratio in animals treated with transporter substrate or inhibitor can be used by microdialysis to confirm possible drug-BBB transporter interactions.

BBB permeability is changed in various brain disorders such as epilepsy, ischemia or hemorrhagic stroke, and malignant gliomas. Genetically modified mice models of brain tumors are examples of preclinical models that replicate these illnesses. These models have progressed from constitutive tumor suppressor gene inactivation to inducible changes for increased specificity. Despite the rise of patient-derived xenograft models, genetically modified mice and immunocompetent rodent models remain essential for preclinical therapy testing in brain tumor research. Advanced techniques further aid drug brain-tumor exposure enhancement. These in vivo models offer critical insights into drug transport across the BBB under various pathological conditions, contributing to developing effective CNS drug delivery strategies [4].

Exploring BBB functionality with in situ models

In certain scenarios, obtaining additional insights beyond conventional in vivo data becomes essential for understanding the mechanisms that hinder drug entry into the brain through the BBB. Researchers cannot control brain blood flow, free drug concentration, or block transport and metabolic processes using the traditional in vivo method. The BBB and other CNS sites that express the key BBB transporters, such as Pgp, organic acid-transporting polypeptide, BCRP, and multidrug resistance protein (MRP) 4, are affected by the genetic modifications of the knockout animals, despite the fact that these animals are available. This complicates the assessment of the BBB-specific contribution to the overall distribution of compounds within the brain. In lieu of direct in vivo analyses, alternative methods such as brain efflux index and in situ perfusion assays offer more targeted investigations into BBB transport mechanisms. These methods complement the standardized intravenous administration approach, providing greater flexibility to study factors influencing transport dynamics. The in situ perfusion technique capitalizes on the native structure of the BBB and cerebral tissues by introducing a specialized vascular perfusion fluid directly into the brain, replacing the circulating blood of the animal. One notable advantage of this method is the ability to precisely manipulate the concentration of solutes in the perfusate, allowing researchers to modulate variables across a broader range than what is typically feasible in vivo [5].

Challenges of BBB

**Figure 2:**
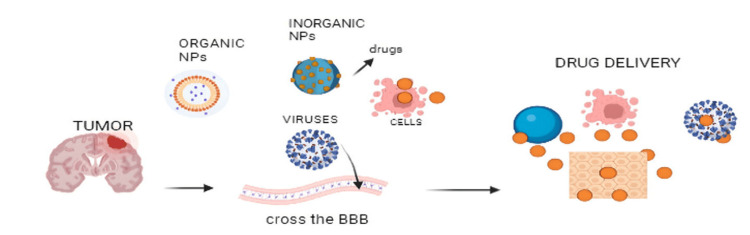
Formulation and challenges of BBB NPs: nanoparticles; BBB: blood-brain barrier Image credit: This image is the original work of the author Sameenkhan Anwarkhan

Addressing the formidable challenges associated with delivering therapeutic compounds to the brain requires a deep understanding of the intricate biological mechanisms governing the BBB and other physiological barriers within the CNS. Here, we delve into the key challenges hindering effective brain targeting. Figure 2 illustrates the various chemical components and nanoparticles attempting to cross the BBB to reach the brain.

BBB Impermeability

The BBB is the principal gatekeeper guarding the CNS, selectively permitting the passage of essential nutrients while restricting the entry of potentially harmful substances. However, this formidable barrier poses an important obstacle to delivering therapeutics to the brain, as it limits the permeability of drugs and therapeutic agents. Overcoming this impermeability barrier is essential for enabling effective treatment of various neurological disorders.

Biological Barriers and Clearance Mechanisms

Beyond the BBB, the CNS harbors additional biological barriers, including the BCSFB and the glymphatic system, each with its unique role in regulating molecular transport within the brain. Moreover, various clearance mechanisms, such as enzymatic degradation and efflux transporters, further impede the effective delivery and retention of therapeutic agents in the brain. Overcoming these barriers necessitates innovative strategies that can bypass or mitigate their effects [6].

Lack of Target Specificity

Achieving precise targeting of therapeutic agents to specific regions or cell types within the brain remains a formidable challenge. Nonspecific distribution might result in reduced treatment effectiveness and off-target consequences. Developing strategies to enhance target specificity while minimizing systemic exposure is critical for optimizing therapeutic outcomes [6].

Drug Stability and Pharmacokinetics

Ensuring the stability of drugs and therapeutic agents during transit through biological barriers is paramount for maintaining their efficacy upon reaching the brain. Factors such as enzymatic degradation and efflux transporters can compromise drug stability and alter pharmacokinetic profiles, thereby limiting therapeutic efficacy. Overcoming these challenges requires the development of drug-delivery systems capable of protecting therapeutic agents from degradation and enhancing their bioavailability within the CNS [6].

Limited Drug Permeability Across BBB

Many promising drugs, particularly large molecules like proteins and nucleic acids, face significant hurdles in crossing the BBB. This limited permeability restricts their therapeutic potential for treating CNS disorders. Innovative approaches aimed at enhancing drug penetration across the BBB hold promise for overcoming this barrier and expanding the repertoire of therapeutics available for neurological conditions [6].

Neuroinflammatory Response

Inflammatory processes within the brain, commonly observed in neurodegenerative diseases, can exacerbate BBB dysfunction and hinder drug delivery. Neuroinflammation induces changes in barrier permeability, further complicating the effective delivery of therapeutic agents to the brain. Strategies aimed at modulating neuroinflammatory responses and restoring BBB integrity are essential for enhancing drug delivery and improving treatment outcomes in neurological disorders [6].

Strategies to improve brain targeting

Enhancing Nasal Drug Delivery to the Brain: Strategies and Innovations

Overcoming the BBB poses an important challenge in drug delivery to the brain. To address this hurdle, researchers have explored various strategies that are broadly classified into invasive and noninvasive approaches. Invasive techniques involve direct intervention at the BBB or brain parenchyma, while noninvasive methods aim to enhance drug delivery without invasive procedures.

Invasive strategies are less common due to their associated discomfort and inconvenience. However, they remain crucial in certain pathologies where other options are limited. Direct administration of drugs or controlled-release systems into the brain parenchyma has been investigated for conditions such as cancers, stroke, and neurological disorders. Controlled-release systems, implanted during surgery, offer sustained drug release over extended periods, enabling long-term treatments. For instance, Food and Drug Administration (FDA)-approved carmustine implants (Gliadel®, Eisai Inc., Tokyo, Japan) have shown efficacy in recurrent glioblastoma treatment by releasing the drug over several days postimplantation [7].

Injection into the CSF is another invasive approach, although it lacks efficient drug diffusion between CSF and extracellular fluid. While more accessible than brain parenchymal injection, CSF injection is utilized in certain infectious diseases like meningitis.

Noninvasive strategies offer alternatives to invasive procedures, aiming to enhance drug delivery efficiency without direct intervention at the BBB. The nasal-to-brain route, for example, leverages the olfactory and trigeminal nerve pathways to bypass the BBB, facilitating direct drug delivery to the brain. Efflux transporter inhibition at the BBB prevents drug expulsion, enhancing drug retention and availability in the brain.

Chemical modifications, such as prodrugs and chemical drug delivery systems (CDDS), enhance drug properties to facilitate BBB penetration and brain uptake. Additionally, nanocarriers have emerged as promising vehicles for drug delivery, enhancing drug solubility, stability, and targeting specificity, thereby facilitating BBB penetration and brain accumulation [7].

Enhancing BBB Permeability: Therapeutic Approaches

Another invasive technique to enhance CNS access involves therapeutically opening the tight bond in the BBB. This can be achieved through the administration of hyperosmolar solutions or the use of ultrasounds. Hyperosmolar solutions, usually formulated with mannitol or other aromatic compounds, cause endothelial cells to expel water, shrinking their size and widening the gaps between them. However, this approach is reserved for life-threatening diseases due to the nonbiased opening of the BBB, which allows both toxic substances and drugs to attain the CNS, potentially causing neurological difficulties such as hemiparesis and aphasia. Additionally, studies administering mannitol with penetration markers have shown the nonuniform distribution of BBB disruption, with varying permeability rates observed depending on the brain region analyzed.

Using ultrasounds in conjunction with the delivery of microbubbles, tiny particles containing heavy gases, is a more focused and selective method. Microbubbles are directed to specific brain areas using ultrasounds, where they converse with endothelial cells to interfere with TJs, allowing drugs to access the BBB freely [6]. Importantly, microbubbles can be filled with drugs or externally altered to carry them, providing a dual therapeutic approach. This method offers the benefit of selectively opening desired areas of the BBB with reduced ultrasound energy requirements.

The use of microbubbles and ultrasounds together has demonstrated the potential for enhancing access of monoclonal antibodies (mAbs) to the CNS, especially when it comes to focusing on specific locations and boosting the immune system to fight illnesses. Various mAbs, when combined with this strategy, have demonstrated efficacy in treating brain cancer and neurodegenerative disorders [7].

*Exploring* *the Transport of Drugs via the Nose to the Brain*

The olfactory region of the nasal cavity presents a viable alternative pathway for delivering chemicals to the neuroaxis. In contrast to other pathways, the olfactory neurons bypass the BBB and connect the nasal passage directly to the neuroaxis. Two possible routes are thought to exist for medications delivered via the nasal passage to reach the brain: trigeminal nerve transportation or olfactory nerve transportation, the latter occurring after absorption from the nasal mucosa.

For example, using insulin through nasal administration has shown potential in Alzheimer’s disease (AD) treatment. Research indicates that when insulin is administered intranasally, it can be found in the CSF but not in the bloodstream. This is because insulin does not easily bypass the BBB due to the BBB's highly selective nature, which restricts the passage of most molecules from the bloodstream into the brain. It has been demonstrated that this technique can improve cognitive abilities in Alzheimer's patients. However, a recent clinical trial involving 289 patients did not detect functional or cognitive benefits following 12 months of intranasal insulin administration. This underscores the necessity for continued refinement and development of intranasal delivery devices.

In the treatment of migraines, intranasal administration has been extensively studied. Trudhesa®, the latest device accepted by the FDA for migraine medication, delivers dihydroergotamine mesylate directly to the upper portion of the nasal cavity (Drugs.com, 2021; NeuroPharma®, 2021, Aminabad, India). A phase 3 safety study revealed rapid pain relief starting just 15 minutes after injection, with relief lasting up to two days after a single dose (Drugs.com, 2021; NeuroPharma®, 2021). This nose-to-brain route offers a promising avenue for drug delivery to the CNS, with potential applications in various neurological disorders [6].

Inhibiting Efflux Transporters: A Strategy to Enhance BBB Permeability

Pgp, BCRP, and the MRP protein family are examples of efflux transporters that are vital for clearing the CNS of metabolic waste products and potentially dangerous substances.

However, when the drug of interest is a substrate of these transporters, it can hinder the medication of brain or spinal cord pathologies. One approach to overcome this challenge is to co-administer the drug with an efflux transporter inhibitor specific to the substrate. However, caution must be used since blocking efflux transporters might cause the influx of xenobiotics into the CNS, resulting in undesirable side effects [6,7].

Over the years, industries have invested in the growth of efflux transporter inhibitors, particularly focusing on Pgp. These inhibitors can be categorized into three generations. The first generation includes molecules like verapamil, quinidine, and cyclosporin A, initially developed for other pathologies but showing some cytotoxicity due to their low specificity for Pgp, with nonspecific interactions with other enzymes and transporters often leading to unplanned adverse effects. To address these limitations, second-generation inhibitors, such as dexverapamil and valspodar, were developed with modifications to enhance potency and reduce pharmacological effects. Despite improvements, these still lack selectivity for Pgp and may interact with metabolic enzymes, leading to negative consequences. The third generation, developed using quantitative structure-activity relationships and computational methods, includes novel compounds like tariquidar, laniquidar, and zosuquidar. These inhibitors have low interactions with other carriers or metabolic enzymes, being specially made to target Pgp. However, clinical trials have shown unanticipated negative consequences during their assessment [8].

Efflux transporter inhibitors have shown promise in treating CNS infections, such as human immunodeficiency virus (HIV). Namanja-Magliano et al. evolved a homodimer of an antiretroviral drug, azidothymidine, capable of inhibiting both Pgp and ATP-binding cassette transporter subfamily G member 2 efflux transporters. Although this homodimer holds the potential for enhancing drug delivery across the BBB by blocking two transporters simultaneously, further research is warranted to validate its efficacy, particularly in clinical settings.

Chemical Strategies: CDDS and Prodrugs

Chemical alteration of molecules represents a versatile tactic not only for enhancing the efficacy of efflux transporter inhibitors but also for developing drug candidates with improved BBB penetration capabilities. Prodrugs are created by modifying an active molecule chemically to increase its lipophilicity. When the prodrug crosses the BBB, it loses its additional piece and becomes an active molecule that may start working. L-dopa, an inactive precursor of dopamine used in the medication of Parkinson's disease, is a well-known example of a prodrug in CNS pathology [7].

Conversely, chemical modifications aimed at attaching a bioremovable targeting structure to a drug lead to the creation of CDDS. Compared to prodrugs, the activation route for drugs from CDDS is more intricate, allowing for the accumulation of intermediary molecules within the brain parenchyma post-BBB penetration, a strategy known as the "lock-in" strategy. For example, the formation of a CDDS by linking dihydrotrigonelline to a drug operates in three phases: first, dihydrotrigonelline enhances the drug's lipophilicity, facilitating its BBB crossing; upon BBB penetration, the system undergoes oxidation, yielding a positively charged molecule that prevents its backflow to the bloodstream; and finally, esterases catalyze the hydrolysis of the intermediary molecule, gradually releasing the active drug. CDDS has a long history, with early studies dating back to 1989, where dihydropyridine was conjugated to penicillinase-resistant penicillins to enhance BBB permeability. Biodistribution tests in rats and rabbits demonstrated detectable CNS levels of penicillins after chemical modification, contrasting with their administration in their native form.

Nanocarriers

Nanocarriers, characterized by their small size ranging from 1 to 100 nm, have emerged as promising vehicles for enhancing drug delivery to the CNS. Their effectiveness stems from their ability to shield drugs from enzymatic degradation, improve plasma stability and solubility, and enable targeted delivery, thereby minimizing undesired side effects. However, for optimal performance, nanocarriers must ensure the controlled release of their loaded drug, necessitating the incorporation of specific features.

First, optimal nanocarriers for CNS delivery should incorporate two distinct ligands: one facilitating BBB penetration and another directing the carrier to a specific brain region. Additionally, they should feature a responsive system triggered by factors like pH or enzymes that swiftly release the drug upon reaching the target site while preventing premature leakage during transit.

Liposomes, a type of nanocarrier, have garnered considerable attention for CNS therapy due to their versatility in encapsulating both lipophilic and hydrophilic drugs within their lipid bilayers and inner cores. They can be categorized into three generations based on their complexity: (1) first-generation liposomes consist solely of a lipid bilayer, making them prone to aggregation and rapid elimination by the reticuloendothelial system; (2) second-generation liposomes, also known as stealth liposomes, feature a phospholipid bilayer surrounded by polyethylene glycol, which enhances stability and reduces immune recognition; and (3) third-generation liposomes, the most intricate, incorporate polyethylene glycosylation like second-generation liposomes but additionally possess other moieties for targeting purposes [7].

Studies have extensively explored third-generation liposomes for various CNS pathologies. For example, liposomes multifunctionalized with apolipoprotein-E (ApoE) and phosphatidic acid (PA) have demonstrated potential in AD therapy. ApoE enhances penetration through the BBB, while PA directs the liposomes to β-amyloid (Aβ) plaques, promoting their dissolution. Moreover, liposomes functionalized with ApoE and PA exhibited enhanced BBB permeability in vitro and higher brain accumulation in healthy mice compared to conventional liposomes.

In another study, liposomes loaded with docetaxel and designed with an ascorbic acid-thiamine disulfide system demonstrated potential for glioblastoma treatment. These liposomes exhibited a "lock-in" behavior post-BBB penetration, resulting in increased brain accumulation of docetaxel in mice compared to the free drug.

Solid Lipid Nanoparticles

Solid lipid nanoparticles (SLNs) are composed of a lipid matrix, making them particularly suitable for delivering hydrophobic drugs. In 2019, He et al. studied SLNs loaded with β-elemene, a naturally occurring essential oil with anti-tumor capabilities, and made with glyceryl monostearate and glycerol tristearate to treat glioblastoma. The findings revealed that SLNs loaded with β-elemene achieved elevated concentrations in both plasma and the brains of mice and rats. This suggests enhanced BBB permeability of β-elemene with this formulation [9].

Further studies, including those administering plain SLNs in vivo, have demonstrated the inherent ability of SLNs to enhance drug penetration across the BBB. Additionally, functionalized SLNs have shown promising outcomes in various studies. For instance, SLNs loaded with quinine dihydrochloride and conjugated with transferrin, aimed at treating cerebral malaria, demonstrated enhanced brain uptake compared to the free drug in solution, attributed to transferrin's facilitation of receptor-mediated transport. Similarly, cationic bovine serum albumin has shown potential as a ligand for functionalizing SLNs to bypass the BBB through adsorptive transcytosis, where the positively charged albumin interacts with the negatively charged endothelial cell surface, facilitating penetration.

Lipid Nanocapsules

Lipid nanocapsules represent another class of lipid-based nanocarriers, offering advantages such as greater stability compared to liposomes and the ability to encapsulate larger quantities of lipophilic drugs. These nanocapsules, which have an oily core surrounded by a strong polymer or surfactant membrane, resemble lipoproteins.

In a study conducted by Elhesaisy and Swidan in 2020, it was demonstrated that lipid core nanoparticles containing trazodone hydrochloride shorten the period of immobility in mice put through a stressful swimming test.

The group treated with nanocapsules exhibited a significant reduction in immobility time compared to the untreated control group and the group treated with a solution of free trazodone. This promising outcome suggests the potential of these carriers as an alternative for managing depression.

Between May 2018 and April 2020, the Bioelectronics or Biological Electronics project concentrated on creating lipid nanoparticles designed to provide antioxidant effects for managing and preventing post-stroke complications (EU Publications Office, 2020, Luxembourg). Current treatments for ischemic stroke, such as tissue plasminogen activator administration or thrombectomy, can restore blood flow but fail to prevent brain tissue damage caused by both nitrogen species release and reactive oxygen. Preliminary findings indicate that the new carrier developed by the project can target both the BBB and neuronal cells [10].

Polymeric Nanoparticles

Polymeric nanoparticles are a versatile category of nanocarriers with promising roles in CNS drug delivery. Based on their structure, they are typically classified into two main groups: nanospheres, which are composed of a solid polymeric matrix, and nanocapsules, characterized by an inner core surrounded by a polymeric shell. Several biodegradable and biocompatible polymers, including chitosan, polycaprolactone (PCL), polylactic acid (PLA), and polylactic-co-glycolic acid (PLGA), have been studied for the creation of tailored nanocarriers for the CNS.

PLA nanoparticles have demonstrated effectiveness in treating fungal meningitis. In a 2015 study, polyethylene glycosylated (PEGylated) PLA nanoparticles loaded with amphotericin B and designed with an anti-transferrin target antibody showed significant efficacy in reducing brain tissue necrosis and improving the survival rate of mice with fungal meningitis.

PLGA nanoparticles have been studied for AD treatment. Barbara et al. engineered PLGA nanoparticles loaded with curcumin and enhanced with a peptide ligand to facilitate BBB penetration. In vitro studies showed that these nanoparticles exhibited promise in reducing Aβ plaque formation and alleviating AD-related inflammation [11].

Chitosan, a natural polysaccharide derived from chitin, has garnered attention for its potential in CNS drug delivery, particularly in Parkinson's disease and AD. When injected intravenously, chitosan-loaded nanoparticles containing rotigotine have improved motor symptoms and enhanced drug accumulation in the brain. These findings indicate the potential of chitosan nanoparticles in managing Parkinson’s disease. These PEGylated particles were nontoxic and enhanced the PCL nanoparticles coated with a drug called clozapine, an antipsychotic medication used to treat schizophrenia. The particles have shown promising results in in vitro experiments assessing the permeability of clozapine across cell monolayers. However, further in vivo studies are needed to test their efficacy before clinical translation [11].

Inorganic Nanoparticles: Advanced Tools for Theragnostic Applications in CNS Disorders

Inorganic nanoparticles, characterized by their composition of metals, metal oxides, or silica, represent cutting-edge modalities poised at the forefront of theragnostic interventions for CNS pathologies. Yet, their utilization is not devoid of intricacies, with their nonbiodegradability and potential for cytotoxicity posing substantial challenges in translational endeavors.

Gold nanoparticles, endowed with the remarkable property of surface plasmon resonance, exhibit unparalleled optical characteristics, permitting absorption and emission of light across a spectrum of wavelengths contingent upon their nuanced dimensions and morphological attributes. This unique feature renders them ideally suited for multifaceted applications, including photothermal therapy, where incident light energy is adeptly transduced into localized heat. Encouragingly, investigations into the efficacy of gold nanoparticles in preclinical models of glioblastoma have given solid outcomes. The translation of these findings into clinical practice is encumbered by formidable obstacles, necessitating meticulous navigation through the intricate matrix of cerebral structures to attain targeted tumor ablation while mitigating collateral damage to adjacent tissues.

Magnetic nanoparticles, predominantly comprising iron oxides, have garnered considerable attention as potential mediators of thermal therapy for glioblastoma and as indispensable adjuncts to various imaging modalities. Furthermore, their role as facilitators of nanocarrier traversing across the BBB underscores their versatile utility. Notably, magnetoliposomes, harboring an internal magnetic core, have exhibited pronounced enhancements in BBB permeability, as corroborated by seminal studies elucidating their efficacy in preclinical models. Recent endeavors have witnessed the exploration of magnetoliposomal formulations in the realm of brain cancer therapeutics, evincing augmented intracerebral accumulation under the influence of an externally applied magnetic field.

Mesoporous silica nanoparticles (MSNs), distinguished by their expansive surface area, robust cargo loading capacity, and biocompatibility, epitomize a burgeoning frontier in nanocarrier development. Leveraging these attributes, researchers endeavor to surmount the formidable barrier posed by the BBB with judicious functionalization strategies enhancing targeted drug delivery to the brain parenchyma. Encouragingly, functionalized MSNs, including lactoferrin-MSNs and Ri7 antibody-MSNs, have demonstrated pronounced affinities for receptor-mediated transcytosis pathways, culminating in augmented brain-specific drug deposition in preclinical models [11].

Dendrimers: Molecular Architects of Therapeutic Precision

Dendrimers are intricate polymeric macromolecules characterized by their three-dimensional architecture comprising a central core, branching arms, and surface functional groups. They epitomize a paradigm of precision drug delivery systems. The delineation of dendrimer generations, contingent upon the extent of branching, underscores their versatility as molecular scaffolds capable of encapsulating and ferrying therapeutic payloads through biological barriers.

Among the pantheon of dendrimers, polyamidoamine (PAMAM) has emerged as a preeminent protagonist in drug delivery endeavors [6]. In a seminal investigation by Xu et al. in 2016, PAMAM dendrimers laden with doxorubicin were meticulously engineered and subjected to rigorous scrutiny through a battery of in vitro and in vivo assessments targeting glioblastoma. Leveraging the strategic incorporation of borneol and folic acid as surface-modifying agents endowed with the dual functionalities of enhancing BBB permeability and imparting tumor-targeting specificity, respectively, the engineered dendrimers exhibited unparalleled efficacy in combating glioblastoma.

In vitro investigations unveiled the nontoxic nature of the dendrimer formulations toward BBB constituents, concomitant with potent cytotoxicity against glioblastoma cells. Notably, a sustained release profile of doxorubicin was discerned under acidic conditions mimicking the tumor microenvironment, coupled with augmented drug permeation across human brain microvascular endothelial cell monolayers. Subsequent in vivo evaluations corroborated the remarkable therapeutic potential of dendrimer-based drug delivery, as evidenced by enhanced accumulation within the brain and tumor milieu, substantial reduction in tumor volume, and concomitant augmentation in animal survival rates.

In essence, dendrimers epitomize a convergence of precision and efficacy in the realm of drug delivery, holding immense promise as vehicles for targeted therapy in the formidable landscape of CNS disorders. However, their realization as clinically viable entities necessitates sustained interdisciplinary efforts to unravel the complex interplay between structure, function, and therapeutic efficacy, thereby ushering in a new era of precision medicine in neurological therapeutics.

Cyclodextrins: Hydrophilic Heroes of Drug Delivery

Cyclodextrins, cyclic polysaccharides renowned for their hydrophilic exterior and hydrophobic interior, have emerged as stalwart allies in the aqueous delivery of lipophilic drugs. Their unique molecular architecture allows them to correspond with lipid membranes, thereby modulating membrane fluidity and enhancing BBB permeability, thus facilitating drug distribution to the CNS.

A groundbreaking advancement in AD therapy has been realized by synthesizing a novel crocetin-γ-cyclodextrin complex. This innovative formulation, meticulously engineered and evaluated, exhibited remarkable therapeutic potential. In vitro studies underscored its nontoxic nature and adept ability to diminish Aβ levels in the 7PA2 cell line. Pharmacokinetic analyses in rats revealed a substantial enhancement in crocetin bioavailability following intraperitoneal administration of the cyclodextrin complex, with plasma concentrations reaching 43.5 times higher and the AUC escalating by 13.1 times compared to standalone crocetin administration. Notably, biodistribution studies elucidated the complex's ability to traverse the BBB and accumulate within the brain parenchyma postadministration [12].

Quantum Dots: Illuminating Pathways in CNS Theranostics

Quantum dots (QDs), diminutive nanosystems imbued with semiconductor properties, herald a new era in CNS theranostics owing to their unique optical characteristics. Analogous to gold nanoparticles, QDs can emit light across diverse wavelengths contingent upon their size, shape, and composition, thereby positioning them as versatile theragnostic agents.

In the realm of CNS pathology management, QDs have garnered considerable attention for their utility in tumor targeting, ischemia detection poststroke, and treatment of HIV-associated encephalopathy. Using a unique triculture in vitro paradigm, QDs have exhibited extraordinary efficiency in blocking HIV replication inside infected peripheral blood mononuclear cells. This is achieved by leveraging the powerful combination of saquinavir as an antiviral drug and transferrin as a BBB-targeting receptor, thus showcasing their potential as formidable contenders in CNS therapeutics [12].

Nanogels: Softening the Barrier to CNS Drug Delivery

Nanogels, intricate nanoparticles characterized by a cross-linked hydrophilic polymer network, epitomize a promising avenue in drug delivery owing to their remarkable biocompatibility and controlled drug release capabilities. Although their potential for CNS therapeutics remains relatively underexplored, a recent study sheds light on the pivotal role of nanogel stiffness in BBB permeability. Through meticulous experimentation involving the synthesis of four distinct nanogel formulations with varying polymer compositions and polymerization durations, Ribovski et al. unraveled a compelling correlation: nanogels exhibiting lower stiffness profiles manifest enhanced intracellular trafficking and exocytosis across human cerebral microvascular endothelial cells/D3 BBB in vitro models. This paradigmatic shift underscores the significance of soft nanogels as a cornerstone in developing CNS-targeted pharmaceuticals [12].

Nanoemulsions: Navigating the Intricacies of BBB Permeability

Nanoemulsions, dynamic amalgamations of two immiscible liquids, stand at the vanguard of versatile drug delivery platforms capable of ferrying hydrophilic and hydrophobic therapeutic agents. The stability of nanoemulsions is intricately tied to the size of their droplets, with smaller dimensions correlating with heightened stability. They orchestrate BBB permeation through a multifaceted array of mechanisms, including lipid exchange engendered by interactions between nanoemulsion lipid phases and endothelial cell membranes, receptor-mediated transport facilitated by surface ligand decoration, adsorptive-mediated transcytosis empowered by positively charged lipid headgroups, and efflux transport inhibition where nanoemulsion droplets shield drugs from efflux transporters, aided by surfactants like polysorbate 80, a renowned Pgp inhibitor. The versatility of nanoemulsions extends to their application in diverse CNS pathologies, including brain tumors, ischemic stroke, HIV-associated CNS conditions, neurodegenerative disorders, and schizophrenia, underscoring their potential as indispensable tools in the armamentarium of CNS therapeutics [12].

Viral Vectors: Illuminating Pathways in Gene Therapy for Neurological Disorders

In the realm of gene therapy, vectors have emerged as stalwarts for addressing nervous disorders, lauded for their exceptional transfection efficiency and sustained expression capabilities. Among these vectors, the adeno-associated virus serotype 9 (AAV9) stands out as a beacon of promise for CNS gene therapy. Distinguished by its ability to traverse endothelial cells via active transport while preserving the integrity of BBB, AAV9 epitomizes a transformative tool in the neurotherapeutic arsenal.

Clinical endeavors have spotlighted AAV9's potential in treating spinal muscular atrophy (SMA), a debilitating genetic affliction targeting alpha motor neurons in the brainstem and spinal nerve. The insidious destruction of these critical neurons precipitates a spectrum of challenges encompassing motor function, speech, respiration, and swallowing, with infancy mortality rates ominously elevated. Encouragingly, early intervention trials employing AAV9 have yielded auspicious outcomes, exhibiting ameliorated motor behavior and enhanced survival rates among afflicted individuals. However, the journey toward harnessing AAV9 as a definitive therapeutic vector for SMA remains fraught with challenges, including the emergence of immunogenic responses in select patients, which may precipitate adverse effects warranting meticulous scrutiny and further research endeavors [12].

BBB dysfunction across various pathological states

The BBB, which regulates the flow of chemicals between the circulation and the brain, is essential for preserving CNS homeostasis. However, in a multitude of CNS disorders, ranging from multiple sclerosis (MS) to lysosomal storage diseases, the BBB exhibits compromised functionality. This impairment can take many different forms. For example, it can cause short-term changes in BBB permeability due to a narrow junction opening, as well as long-term barrier disruption and structural changes, including basement membrane deterioration.

Damage to surrounding brain tissue results from developing microglia, the influx of immune cells and plasma components into the brain tissue, and disturbance of CNS homeostasis. While the causative role of BBB compromise in disease onset remains elusive in many cases, its contribution to exacerbating pathology is evident.

The BBB keeps a reasonably impermeable barrier in physiological settings. However, in pathological states, releasing cytokines, vasoactive agents, and chemical mediators increases BBB permeability. Key mediators include aspartate, ATP, endothelin-1, glutamate, nitric oxide (NO), taurine, tumor necrosis factor-alpha (TNF-α), and macrophage-inflammatory protein 2, predominantly produced by astrocytes. Additionally, humoral agents such as 5-hydroxytryptamine, bradykinin, thrombin, histamine, uridine monophosphate, uridine triphosphate, substance P, platelet-activating factor, quinolinic acid, and free radicals contribute to increased BBB permeability, sourced from endothelium or nerve terminals associated with blood vessels [13].

Ischemic Stroke

Numerous investigations on the effects of hypoxia-ischemia on the BBB have shown that soluble substances such as NO, cytokines, and vascular endothelial growth factor, also known as vascular endothelial growth, cause increased permeability and abnormalities in TJs. Elevated levels of proinflammatory cytokines such as TNF-α and interleukin (IL)-1β have been observed in animal brains postischemia, exacerbating BBB permeability. Moreover, in vitro investigations have elucidated the secretion of inflammatory mediators by astrocytes under hypoxic conditions, further contributing to BBB dysfunction [13].

Brain Tumors

The compromised BBB in brain tumors, attributed to poorly developed vasculature, presents as disruptions in interendothelial TJs. Alterations in claudin-1, claudin-5, occludin, and zonula occludens 1 expression levels underscore the dysregulation of BBB integrity. Notably, upregulation of the water channel molecule aquaporin-4 correlates with increased BBB permeability in tumor microenvironments, implicating its role in cerebral edema [14].

Metastatic Lesions

Despite the brain's robust barrier to cancerous cells, metastatic lesions from extracranial cancers can colonize the CNS, partly attributed to partial BBB disruption facilitating tumor cell entry. Chemotherapeutic challenges arise due to limited drug permeability through the BBB, necessitating brain-permeable molecular therapeutics to effectively target lesions.

Septic Encephalopathy

Septic encephalopathy's pathophysiology encompasses reduced cerebral blood flow, cerebral edema, oxygen extraction, and disruption of BBB, influenced by diverse factors. Cerebrovascular tiny vessels, the makeup of neurotransmitters in the retinal triggering system, astrocyte activity, and degeneration of neurons, are all impacted by inflammatory mediators. Studies using 14C amino acid, 125I-albumin, and colloidal iron oxide in rodents have demonstrated their entry into the brain parenchyma during septic encephalopathy, indicating BBB breakdown. Cellular diseases that underlie the breakdown of the BBB include swelling of the astrocyte end-feet and enhanced pinocytosis in the cerebral microvessel endothelium. The adrenergic system, particularly β2 adrenoreceptor suppression and α1 adrenoreceptor stimulation, influences inflammatory responses and BBB permeability [14].

HIV Encephalitis

HIV infection of the CNS triggers immunological activation of macrophages and astrocytes, which produces chemokines, cytokines, neurotoxins, and reactive oxygen species. These substances cause leukoencephalopathy, interfere with neurotransmitter activity, and damage cells and neurons. TNF-α, along with neurotoxins like NO, arachidonic acid, quinolinic acid, and platelet-activating factor, contributes to severe neurologic pathologies. HIV-associated dementia correlates with compromised BBB integrity, facilitating viral entry into the brain and exacerbating neuronal injury. Serum protein leakage and TJ protein alterations further characterize BBB dysfunction in HIV encephalitis. The HIV envelope glycoprotein gp120 significantly induces BBB permeability, promotes albumin extravasation, and upregulates cellular and vascular adhesion molecules [15].

Alzheimer’s Disease

In AD, the accumulation of Aβ peptides triggers microglial and astrocytic activation, releasing cytokines and reactive oxygen species, contributing to neuronal damage and synaptic dysfunction. Immune cell activation and cytokine release affect BBB integrity, exacerbating AD pathology. By attaching to low-density lipoprotein receptor-related protein 1, which is present on the BBB abluminal surface, Aβ is transferred from the brain to the blood. Phosphatidylinositol binding clathrin assembly protein (PICALM) facilitates this process. PICALM gene mutations are thought to be hereditary risk factors for AD and may influence the course of the illness. Additionally, the APOE4 mutation, responsible for Aβ clearance, disrupts BBB function and exacerbates AD pathology [15].

Multiple Sclerosis

Reactive T cells engage with antigens delivered by macrophages or microglia displaying HLA-DR2a and HLA-DR2b in MS, an inflammatory disease that causes the myelin sheath and underlying axons to be destroyed. Upon activation, macrophages release cytokines and nitric acids such as IL-3, interferon-γ, and TNF-α. These molecules destroy oligodendrocytes, disrupting myelination and impairing myelin gene function. The BBB disruption represents an early and crucial event in MS, accompanied by significant penetration of T cells and the development of demyelinated lesions. Additionally, MS lesions demonstrate elevated levels of reactive oxygen species, which contribute to brain damage and participate in various pathogenic processes underneath the formation of MS lesions. Increased levels of lipid peroxidation products and NO metabolites have been detected in the serum of individuals with MS [16]. Table 1 provides an overview of BBB dysfunction across a range of pathological conditions, detailing the different states and their impact on the integrity and function of the BBB.

**Table 1:** Pathological conditions and description of BBB dysfunction BBB: blood-brain barrier; AD: Alzheimer's disease; TJs: tight junctions; MS: multiple sclerosis

Pathological condition	Description of BBB dysfunction	Study
Septic encephalopathy	Reduced cerebral blood flow and cerebral edema, oxygen extraction, and blood-brain barrier disruption are consequences of inflammatory mediators, altered neurotransmitter levels, dysfunctional astrocytes, and neuronal degeneration	Kadry et al. [17]
HIV encephalitis	Activation of astrocytes and macrophages by immune responses results in the release of cytokines, chemokines, and reactive oxygen species. This process impairs cellular function and disrupts neurotransmitter action. Additionally, compromised blood-brain barrier integrity facilitates viral entry into the brain	
AD	The buildup of β-amyloid protein and connected oligopeptides triggers the activation of astrocytes and microglia, prompting the release of toxic molecules. This process leads to neuronal damage, synaptic dysfunction, and activation of immune cells, ultimately compromising the BBB	
Ischemic stroke	Increased levels of vascular endothelial growth factor and proinflammatory cytokines result in the interfere with TJs and heightened permeability of the BBB. This condition facilitates the transmigration of leukocytes and contributes to cerebral edema	
Brain tumors	Disruption of interendothelial TJs, downregulation of TJ molecules, and upregulation of aquaporin-4 expression leading to increased vascular permeability and cerebral edema	
MS	The autoimmune-driven devastation of the underlying axons and myelin sheath, coupled with BBB disruption, substantial infiltration of T cells, and increased reactive oxygen species levels, collectively contribute to brain damage	

Quality by design

Successful brain-targeted drug delivery requires a comprehensive understanding of formulation chemistry, pharmacokinetics, and safety considerations. Quality by design (QbD) principles play a crucial role in optimizing drug formulations for effective brain delivery while ensuring safety and efficacy. Integrating QbD approaches with advanced technologies and methodologies can facilitate the expansion of novel drug delivery systems tailored to specific CNS targets. This section explores applying QbD principles to overcome the challenges associated with brain-targeted drug delivery.

Identification of CQAs for Brain Targeting

In QbD, the first step is to define the critical quality attributes (CQAs) of the drug product. For brain-targeted delivery systems, CQAs may include factors such as particle size, surface charge, drug release kinetics, and capacity to pass the BBB [18]. These attributes are crucial for ensuring the drug reaches the desired site of action in the brain with optimal efficacy.

Understanding of CPPs

QbD involves a systematic understanding of critical process parameters (CPPs) that influence the CQAs of the formulation. For brain-targeted drug delivery systems, CPPs may include parameters related to formulation techniques (e.g., nanoparticle fabrication methods), excipient selection, and drug loading strategies [18]. Researchers can optimize the formulation process to achieve desired product attributes by comprehensively studying these parameters.

DOE and Risk Assessment

QbD utilizes the design of experiments (DOE) and risk assessment tools to evaluate the impact of formulation variables on product performance systematically. In brain-targeting articles, researchers may employ DOE to investigate the effects of different nanoparticle formulations, drug payloads, and surface modifications on BBB penetration and therapeutic efficacy [19]. Additionally, risk assessment methodologies help identify and mitigate potential formulation risks that could impact product quality and patient safety.

Real-Time Monitoring and Control

QbD advocates for real-time monitoring and control of critical parameters during formulation and manufacturing processes. In brain-targeted drug delivery, advanced analytical techniques such as in vivo imaging, pharmacokinetic studies, and neuroimaging modalities enable researchers to assess drug distribution, BBB permeability, and therapeutic outcomes in real time [19]. This allows for proactive adjustments to formulation parameters to optimize brain targeting efficiency.

Continuous Improvement and Lifecycle Management

QbD promotes continuous improvement and lifecycle management of drug products. In brain-targeting research, ongoing optimization efforts, informed by preclinical and clinical studies feedback, help refine formulation strategies and enhance therapeutic outcomes [20]. Additionally, postmarketing surveillance ensures that brain-targeted drug delivery systems remain safe, effective, and compliant with regulatory standards over time. In summary, QbD principles provide a systematic framework for expanding brain-targeted drug delivery systems, facilitating the design, optimization, and quality assurance of formulations aimed at treating neurological disorders.

Future perspectives

In the foreseeable future, advances in nanotechnology are poised to greatly improve the accuracy and effectiveness of brain-targeted drug delivery systems, enhancing their capacity to breach the BBB. Customized medical approaches will be pivotal, tailoring therapies to individuals' genetic makeup and specific disease traits, thereby optimizing treatment outcomes and reducing side effects. Implantable devices, such as neural interfaces and drug-releasing implants, will provide enduring solutions for localized drug administration, either autonomously reacting to physiological signals or externally controlled. Bioengineering breakthroughs, encompassing cell-based treatments and genetically engineered viruses, hold potential for innovative strategies in treating neurological disorders, utilizing modified cells as drug producers or viral vectors for targeted gene transport. Noninvasive delivery techniques, like focused ultrasound and magnetic guidance, will offer nonsurgical options, ensuring precise drug delivery with minimal tissue disruption. Integrating these advancements with imaging technologies will enable real-time monitoring of drug dispersal and treatment efficacy, facilitating tailored and efficient therapies for diverse neurological conditions.

## Conclusions

Brain drug targeting faces significant challenges due to the BBB and the brain's complex environment. While nanoparticles and biological therapies hold promise, several gaps remain. Future research should focus on understanding nanoparticle-BBB interactions, exploring long-term biocompatibility, and enhancing targeting efficiency to specific brain regions or cell types. Further study is needed to integrate nanoparticles with biological therapies, such as gene therapy, and to develop personalized approaches based on individual BBB variability.

Improving nanoparticle targeting efficiency and exploring combination therapies with existing treatments are also crucial. Advancements in real-time monitoring and imaging of nanoparticle distribution and drug release within the brain are necessary. Ethical and regulatory considerations must be addressed to ensure the safe clinical translation of these therapies. Collaboration between researchers and regulatory bodies is essential to establish guidelines for nanoparticle-based treatments. Accelerating the transition from preclinical studies to human trials will require interdisciplinary efforts, ultimately enhancing the efficacy and safety of brain drug targeting strategies.
